# Nature the Great Teacher

**Published:** 1886-09

**Authors:** 


### *NATURE THE GREAT TEACHER.*

Rev. J. G. Wood, in his new book, *Nature's Teachings*, has discussed a subject not before handled at length. Its object is to show how man's implements and mechanical devices have been anticipated in nature. He asserts that there is no invention of man which is not anticipated, that all his mechanical devices have been used in nature for countless centuries. He claims that the great discoverers of the future will be those who carefully study the natural world.

The burr-stones of mills are a copy of molar teeth. The hoofs of a horse are made of parallel plates like a carriage spring. The finest file made by man is a rough affair when compared with the Dutch rush used by cabinetmakers. The jaws of the turtle and tortoise are natural scissors. Rodents have chisel teeth, and hippopotami have adz teeth, which are constantly repaired as they are worn. The carpenter's plane is anticipated by the jaws of a bee. The woodpecker has a powerful little hammer. The diving-bell only imitates the work of the water-spider. This insect, although as easily drowned as any other, spends a great part of its life under water. Having constructed a small cell under the water, it clasps a bubble of air between its last pair of legs and dives down to the entrance of its cell, into which the bubble is put. A proportionate amount of water is thus displaced, and when all of it is expelled the little animal takes up its abode in this sub-aqueous retreat.

In laying its eggs on the water, the gnat combines them in a mass shaped somewhat like a life-boat. It is impossible to sink it without tearing it to pieces. The iron mast of a modern ship is strengthened by deep ribs running along its interior. A porcupine quill is strengthened by similiar ribs. When engineers found that hollow beams were stronger than solid ones, they only discovered a principle which had been used in nature for centuries before the creation of man. A wheat straw, if solid, could not support a heavy head. The bones of the higher animals, if solid, would have to be a great deal heavier to bear the weight which they have to support. The framework of a ship resembles the skeleton of a herring, and he who would improve aerial navigation might study the skeleton of a bird with advantage. Palissy made a careful study of the shells by the seaside, in order to learn the best method of fortifying a town.

The ship-worm feeds on wood, and gradually tunnels its way through any submerged timber. It also lines its burrow with a hard, shelly coating. Brunel, taking a hint from this, was the first to succeed in subaquatic tunneling. The Eddystone Light-house is built on the plan of a tree-trunk, and fastened to the rock in a manner somewhat similiar to the way a tree is fastened to the soil. It is supposed that the first idea of a suspension bridge was suggested by the creepers of a tropical forest.

Mr. Wood gives an interesting account of the origin of the plan for the Crystal Palace. Mr. Paxton, a gardener, having noticed the structure of the great leaves of the Victoria Regia, a plant which had been introduced into England a few years previous, struck the plan of copying in iron the ribs of the leaf and filling the remaining space, which corresponds to the cellular portions of the leaf, with glass. Thus, by copying nature, an obscure gardener became Sir Joseph Paxton, the great architect.—*Wilmington Collegian*

